# Differential Behavior within a Grapevine Cluster: Decreased Ethylene-Related Gene Expression Dependent on Auxin Transport Is Correlated with Low Abscission of First Developed Berries

**DOI:** 10.1371/journal.pone.0111258

**Published:** 2014-11-03

**Authors:** Nathalie Kühn, Carlos Abello, Francisca Godoy, Serge Delrot, Patricio Arce-Johnson

**Affiliations:** 1 Departamento de Genética Molecular y Microbiología, Pontificia Universidad Católica de Chile, Santiago, Chile; 2 Université de Bordeaux, ISVV, INRA, EGFV, UMR 1287, Villenave d'Ornon, France; National Taiwan University, Taiwan

## Abstract

In grapevine, fruit abscission is known to occur within the first two to three weeks after flowering, but the reason why some berries in a cluster persist and others abscise is not yet understood. Ethylene sensitivity modulates abscission in several fruit species, based on a mechanism where continuous polar auxin transport across the pedicel results in a decrease in ethylene perception, which prevents abscission. In grapevine, flowering takes about four to seven days in a single cluster, thus while some flowers are developing into berries, others are just starting to open. So, in this work it was assessed whether uneven flowering accounted for differences in berry abscission dependent on polar auxin transport and ethylene-related gene expression. For this, flowers that opened in a cluster were tagged daily, which allowed to separately analyze berries, regarding their ability to persist. It was found that berries derived from flowers that opened the day that flowering started – named as “first berries” – had lower abscission rate than berries derived from flowers that opened during the following days – named as “late berries”. Use of radiolabeled auxin showed that “first berries” had higher polar auxin transport, correlated with lower ethylene content and lower ethylene-related transcript abundance than “late berries”. When “first berries” were treated with a polar auxin transport inhibitor they showed higher ethylene-related transcript abundance and were more prone to abscise than control berries. This study provides new insights on fruit abscission control. Our results indicate that polar auxin transport sustains the ability of “first berries” to persist in the cluster during grapevine abscission and also suggest that this could be associated with changes in ethylene-related gene expression.

## Introduction

Survival within a community depends on several genetic and environmental factors that define which organisms will be able to maintain their reproductive cycle, while others stop their development. Within a single organism, this notion may also be applied to determined cells or tissues that are predestined to survive. What does determine this successful status, is an interesting question not always easy to solve. In plants, it is possible to consider some naturally occurring processes as selection mechanisms. For instance, many fruit species that produce abundant flowers are not able to support the growth of all fruits, and some of them are selected to continue their growth, with fruit abscission the selection mechanism involved. Fruit abscission, described as the physiological drop of fruitlets, allows to define how many fruits will persist, ensuring an adequate destination of photosynthates, water and ions. In fact, plants bearing a heavy fruit load per cluster show higher fruit abscission rates compared to plants with a small number of fruits [1], indicating that there is a control of the fruit load and that abscission has a key role.

In plants, a set of hormones is responsible for the execution of specific responses. Since multiple and complex processes must be regulated, combinatorial interactions between hormones are required [2]. Antagonistic effects of ethylene and auxin have been reported for the abscission of flowers and fruits [3]. While ethylene promotes abscission in several fruit species [4]–[6], auxin on the other hand prevents abscission by retarding the activation of the so-called abscission zone (AZ) at the fruit pedicel [1]. For instance, it has been shown that the formation of the AZ in the pedicel of tomato (*Solanum lycopersicum*) fruitlets in response to auxin depletion by flower removal is reversed by the application of indole-3-acetic acid (IAA) to the cut surface of the pedicel [7]. Auxin and ethylene crosstalk controls fruit abscission through a mechanism involving modulation of ethylene sensitivity by polar auxin transport. Thus, maintaining a low level of ethylene sensitivity requires a constant polar supply of auxin to the pedicel AZ [8]. This supply comes from the developing fruit [9]–[11]. So, if the source of IAA is removed and the auxin gradient is altered, the AZ becomes sensitive to ethylene and abscission occurs [1], [12], [13]. At the molecular level, it has been shown that chemically induced abscission in apple (*Malus domestica* L.) fruitlets correlates with an increase in the transcript abundance of genes associated with ethylene perception and biosynthesis [14]. In tomato fruitlets, prior to and during pedicel abscission, an increase in the transcript abundance of genes coding for auxin and ethylene transcription factors has been reported [7].

Abscission depends on many environmental and internal cues. Among internal cues, interaction between fruitlets is especially important, since it determines that some fruits – “dominant fruits” – develop earlier and have lower abscission rate compared to the other fruits – “dominated fruits” – and also exert a negative effect on them, which are more prone to abscise [1]. Interestingly, “dominated fruits” show lower auxin export than “dominant fruits”, and this is abolished by removal of dominant fruits [15]. Comprehensive molecular characterization of this phenomenon is scarce. The most recent example is a global expression analysis in which big apple fruitlets were compared with small young fruits [16]. It was found that gene expression related to abscisic acid and ethylene signaling pathways were induced while that of gibberellin was down-regulated in small fruitlets chemically induced to abscise, compared with big fruitlets that were aided to persist by removing lateral fruitlets of the cluster. However, in this work, neither the connection between auxin flux and fruitlet abscission nor the molecular events associated with changes in fruitlet dominance due to polar auxin transport inhibition were assessed [16].

Grapevine (*Vitis vinifera* L.) berries are non-climacteric fleshy fruits disposed in a cluster of several dozens of berries [17]. During the first two weeks of development, an abrupt increase in berry size due to cell multiplication and, to a lesser extent, to cell enlargement and occurs, which is known as fruit set [18], [19]. During this time abscission occurs as well [20], [21], correlating with an increase in ethylene content [4]. It has been shown that exogenous application of ethylene causes berry drop, demonstrating the role of this hormone in grapevine berry abscission [22], [23]. On the other hand, it is not known whether auxin, which is highly abundant in young berries [24], is involved in the control of ethylene sensitivity during grapevine berry abscission. Considering that transgenic vines containing high IAA amounts in the ovules at fruit set produce more berries per cluster [25], it may be hypothesized that auxin could prevent berry abscission. Nevertheless, it is not known whether constant auxin export from the fruitlets leads to lower sensitivity to ethylene and hence to decreased abscission in grapevine berries.

In grapevine, flowering takes about four to seven days in a single cluster; thus, while some flowers are already developing into fruits, others are just opening in the same cluster. Hence, the aim of this work is to determine whether uneven flowering, resulting in berries with dissimilar developmental status, accounts for differences in abscission, and whether this is correlated with changes in polar auxin transport and ethylene-related gene expression. Although the connection between fruitlet abscission and polar auxin transport has been previously investigated, to date abscission variations associated with flowering time have not been assessed. This work provides new insight into the regulation of fruitlet abscission.

## Results

### Abscission and polar auxin transport in “first berries” compared to “late berries”

**Figure 1:**
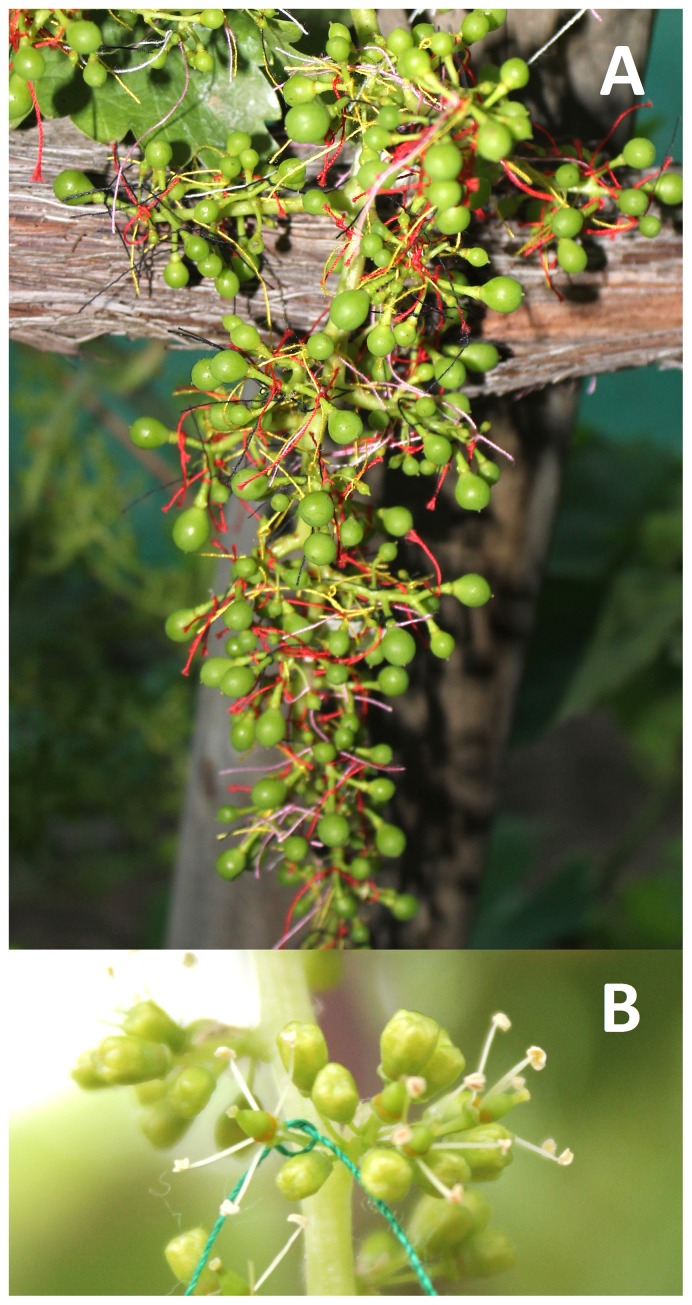
Berry categories identified according to flowering time. (A) Berry tagging with colored threads allowed to differentiate four categories within a grapevine cluster depending on the day the flowers opened. “First berries” (berry category 1) were those derived from flowers that opened the day that flowering started, *i. e.* day 1. “Late berries” were those derived from flowers that opened during days 2, 3 and 4 (berry categories 2, 3 and 4, respectively). Berry category 4 is also named as “last berries. Picture was taken 21 days after flowering (DAF), with 0 DAF the day when the first flower opened in the cluster. (B) Tagging was performed daily on flowers that had just open by putting colored threads around the pedicel.

**Figure 2:**
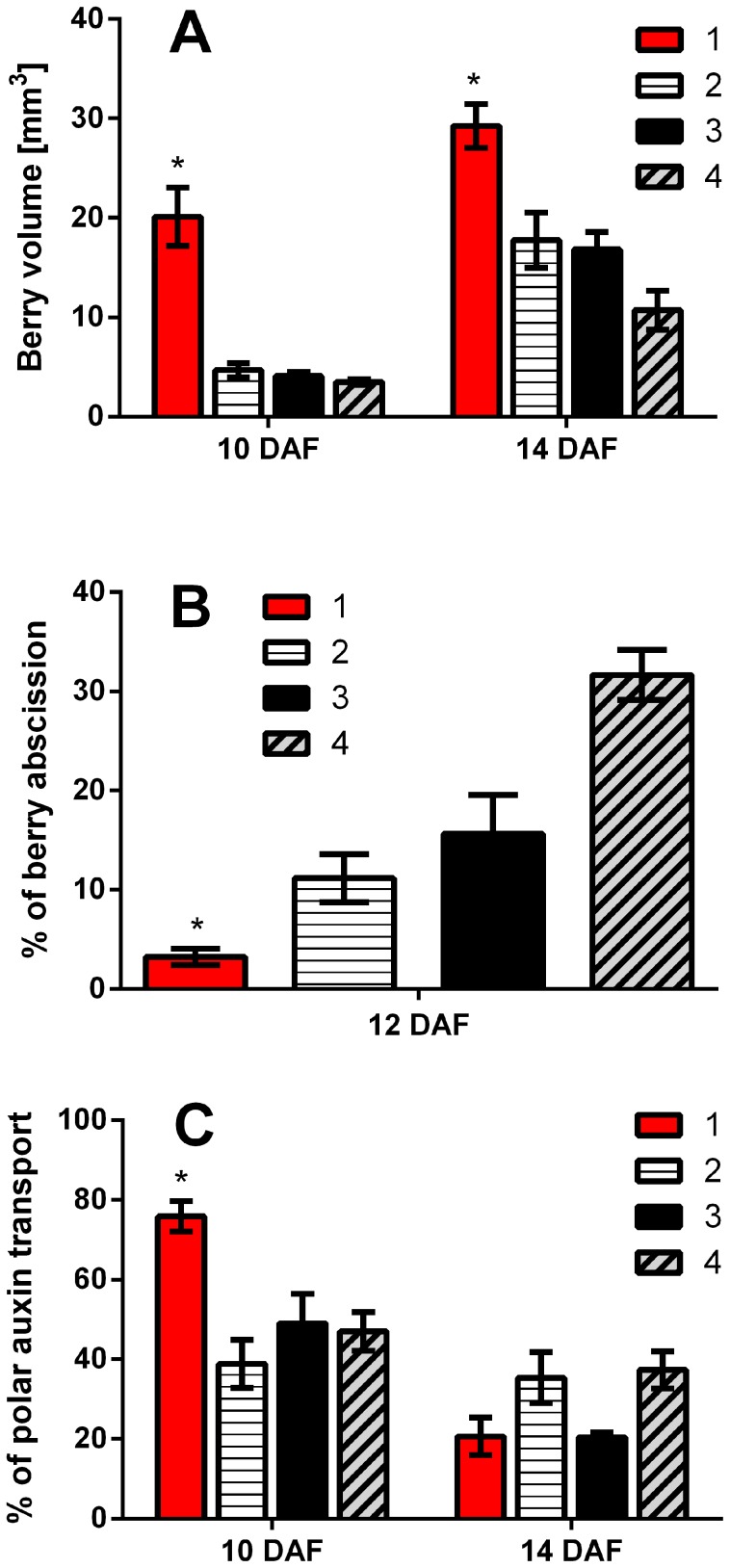
Berry size, abscission rate and polar auxin transport in “first berries” compared to “late berries”. (A) Berry volume differences between berry categories 1, 2, 3 and 4 assessed at 10 and 14 DAF. (B) Abscission differences between berry categories 1, 2, 3 and 4 assessed at 12 DAF relative to 8 DAF (for berry number see Table 1). (C) Basipetal auxin transport differences between berry categories 1, 2, 3 and 4 assessed at 10 and 14 DAF. Asterisk indicates that “first berries” are significantly different from “late berries”. Significance was assessed using p value <0.05 (n = 3). Error bars are STERR±. Comparisons were established between same age berries.

Four berry categories were indentified within grapevine clusters using a tagging system (Figure 1A). For this, colored threads were tied around the pedicel the day the flowers opened (Figure 1B). This strategy allowed us to connect the berries to their flowering day and developmental status, with “first berries” those derived from flowers that opened the day that flowering started and “late berries” those derived from flowers that opened later on during the three following days. Berry volume measurements showed that size differences between berry categories can be detected as early as 10 days after flowering (DAF; Figure 2A). Although there were volume variations between first and late formed berries at 14 DAF, the most pronounced size differences were observed at 10 DAF. Note that berries were compared at the same developmental stage, *i. e.* the same time passed between bloom and the measurements. Interestingly, “first berries” at 10 DAF showed similar volume than “late berries” at 14 DAF, suggesting that “first berries” develop earlier than “late berries”. It is worth to mention that there were no significant berry volume differences between all categories of “late berries”, at both 10 and 14 DAF (Figure 2A). Therefore, the flowering order between “late berries” is not associated with size differences, but is crucial for volume variations observed between first and late formed berries.

Grapevine berry abscission occurs the first two to three weeks after flowering [20], [21], and is particularly marked around fruit set, the time when an abrupt increase in berry size takes place [18]. When each category of berries was separately analyzed for their ability to persist in the cluster, it was found that “first berries” practically did not abscise, while the rest of the berries had abscission values ranging between 10% and 30% at 12 DAF (Figure 2B). Thus, for abscission, as for berry size, “first berries” and “late berries” were significantly different. As polar auxin transport has been connected to abscission, we investigated whether variations in abscission rate correlated with differences in polar auxin transport capacity. In order to measure basipetal auxin transport across grapevine fruitlets, a drop of radiolabeled IAA was added into the apical surface of berries that were arranged on receiver agar discs. After an 8-h transport period, receiver agars discs were collected and accumulated radioactivity was measured. Percentage of total applied radioactivity present in the receiver agar discs was calculated to estimate auxin movement, and equals the percentage of radioactivity in receiver agars divided by the radioactivity in the berries plus in the receiver agars after the 8-h transport period. As expected, “first berries” showed the highest polar auxin transport percentage among all berry categories at 10 DAF (Figure 2C). At 14 DAF, polar auxin transport percentage was lower than at 10 DAF and did not show a clear pattern associated with berry category (Figure 2C). It should be noted that there were no differences in polar auxin transport percentage between “late berries” (Figure 2C).

### Ethylene-related transcript abundance and ethylene content in “first berries” compared to “last berries”

**Figure 3:**
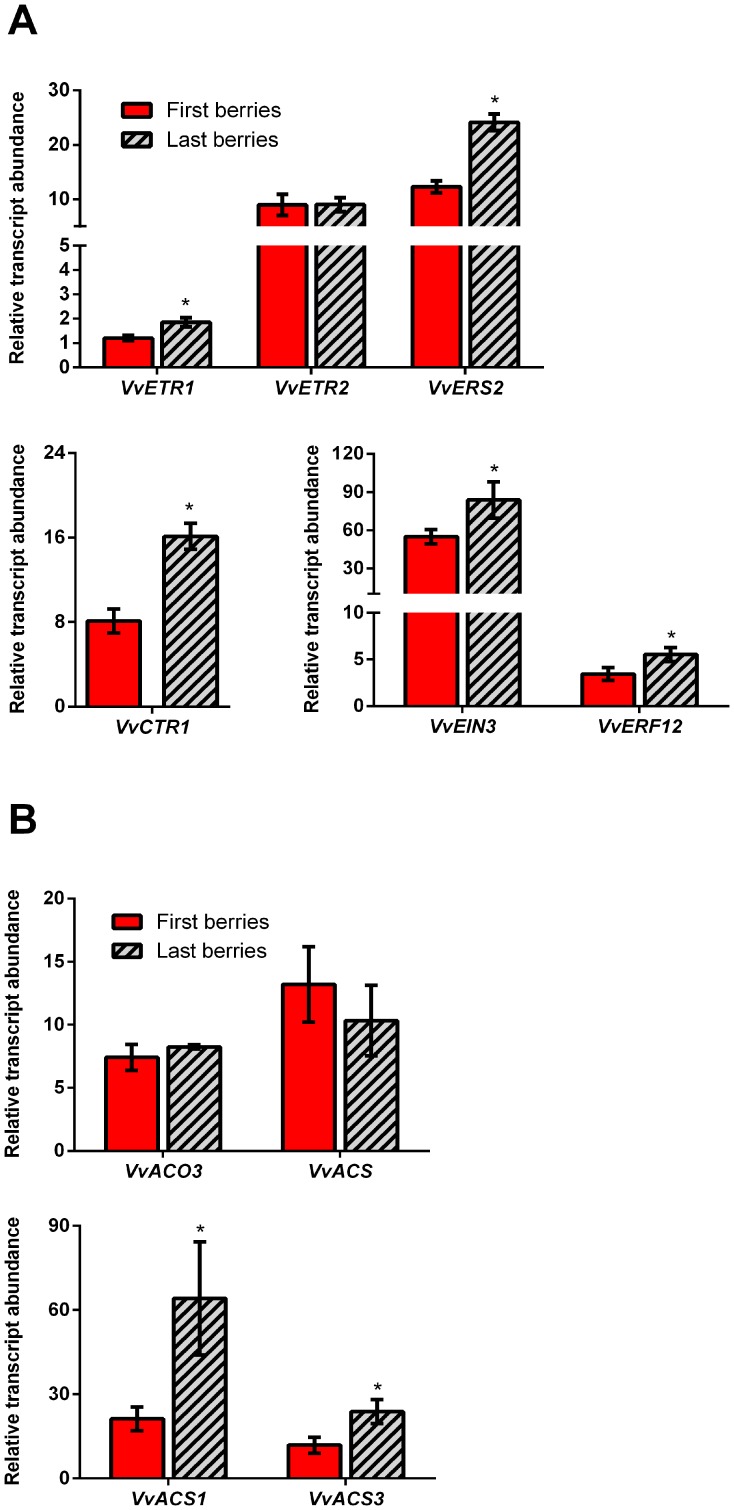
Transcript abundance of ethylene perception- and biosynthesis-related genes in “first berries” compared to “last berries”. (A) Transcript abundance of *VvETR1, VvETR2, VvERS2, VvCTR1, VvEIN3, VvERF12* genes assessed in first and last formed berries at 12 DAF. (B) Transcript abundance of *VvACO3, VvACS, VvACS1* and *VvACS3* assessed on first and last formed berries at 12 DAF. Transcript abundance is relative to the mean expression of the constitutive genes *VvUBI1* and *VvGPDH* (for more details, see Material and Methods section). Significance was assessed using p value <0.05 (n = 3). Asterisk indicates that differences are significant between “first berries” and “last berries” for each gene analyzed. Error bars are STERR±.

It is well known that high polar auxin transport across the pedicel is associated with fruit abscission prevention through an ethylene desensitization mechanism [1], [12], [13]. Therefore, we were interested in determining whether the observed differences in polar auxin transport between berries at 10 DAF could be associated with differences in ethylene-related gene expression. Hence, we compared the relative transcript abundance of ethylene perception- and biosynthesis-related genes between “first berries” and “last berries”, which are those berries derived from flowers that opened the day that flowering ended (Figure 3). The relative transcript abundance of the putative ethylene receptors, *VvETR1* and *VvERS2*, similar to Arabidopsis *ETHYLENE-RESPONSE-FACTOR1* (*ETR1*) and *ETHYLENE-RESPONSE-SENSOR1* (*ERS1*), respectively, was significantly higher in “last berries” than in “first berries”, while transcript abundance of the putative ethylene receptor *VvETR2*, which is similar to Arabidopsis *ETHYLENE-RESPONSE-FACTOR2* (*ETR2*), did not change significantly between “first berries” and “last berries” (Figure 3A). We also found that the relative transcript abundance of the putative negative regulator of the ethylene signaling, *VvCTR1*, similar to *CONSTITUTIVE-TRIPLE-RESPONSE1* (*CTR1*), was significantly higher in “last berries” than in “first berries” (Figure 3A). The relative transcript levels of the putative ethylene transcription factors, *VvEIN3* and *VvERF12*, similar to Arabidopsis *ETHYLENE-INSENSITIVE3* (*EIN3*) and *ETHYLENE-RESPONSIVE-FACTOR12* (*ERF12*), respectively, that could be involved in the transcription control of ethylene responsive genes, were found to be significantly higher in “last berries” than in “first berries” (Figure 3A). These results show that genes possibly involved in ethylene perception are more expressed in “last berries” than in “first berries”. This is associated with the high abscission percentage and low polar auxin transport capacity observed in “last berries”.

**Figure 4:**
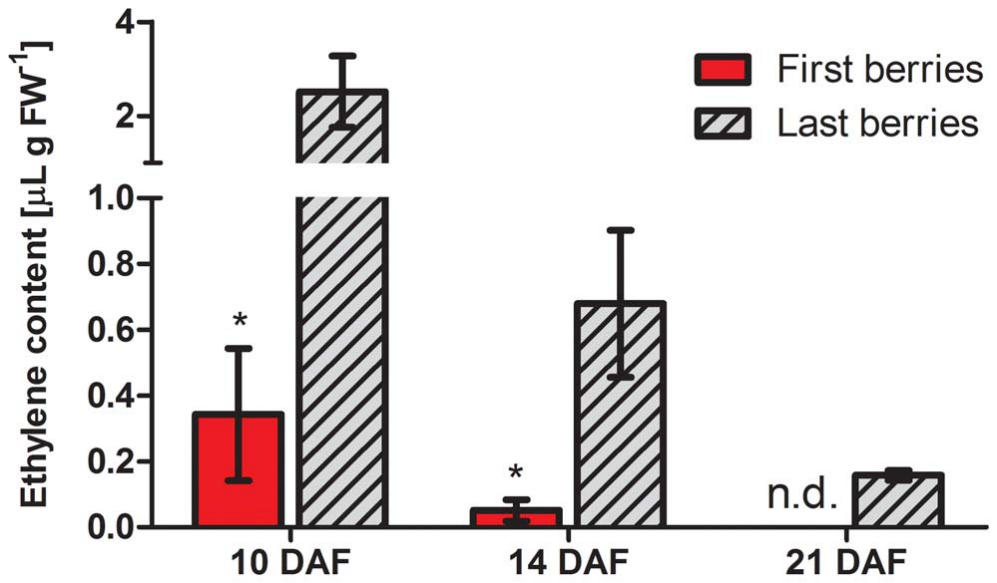
Ethylene content in “first berries” compared to “last berries” during and after the abscission period. Ethylene content in “first berries” and “last berries” measured at 10, 14 and 21 DAF. Asterisk indicates that differences are significant between “first berries” and “last berries”. n.d., not detected. Significance was assessed using p value <0.05 (n = 3). Error bars are STERR±.

A positive feedback allowing amplification of ethylene signal has been reported, in which increased ethylene biosynthesis follows activation of the ethylene response [26]–[28]. Four putative ethylene biosynthetic genes, *VvACS*, *VvACS1* and *VvACS3*, coding for aminocyclopropane-1-carboxylate (ACC) synthases, and *VvACO3*, coding for an ACC oxidase, were found to be expressed at 12 DAF. The transcript levels of *VvACS1* and *VvACS3*, similar to Arabidopsis *1-AMINOCYCLOPROPANE-1-CARBOXYLATE SYNTHASES* (*ACSs*), were significantly higher in “last berries” than in “first berries”. On the other hand, *VvACS* and *VvACO3*, similar to Arabidopis *1-AMINOCYCLOPROPANE-1-CARBOXYLATE OXIDASEs* (*ACOs*), transcript abundance did not change significantly between first and last formed berries (Figure 3B). These results are in agreement with measurements of ethylene evolution in both berry categories from 10 to 21 DAF (Figure 4), showing that “last berries” have higher ethylene content than “first berries” during the abscission time (10 DAF and 14 DAF) and even when abscission was over (21 DAF). Ethylene content in “first berries” at 14 DAF is 0.05 µL per gram of fresh tissue, which is about 6 times higher than reported for grapevine berries at fruit set [4]. Interestingly, in “last berries” ethylene content is more than two orders of magnitude higher, which is correlated with their higher abscission percentage. In summary, low abscission in “first berries” is associated with increased polar auxin transport, reduced transcript abundance of ethylene perception- and biosynthesis-related genes and low ethylene content. The opposite was observed for “last berries”, which are more prone to abscise.

### Effect of disrupting auxin flux in “first berries” on ethylene-related transcript abundance and abscission

**Figure 5:**
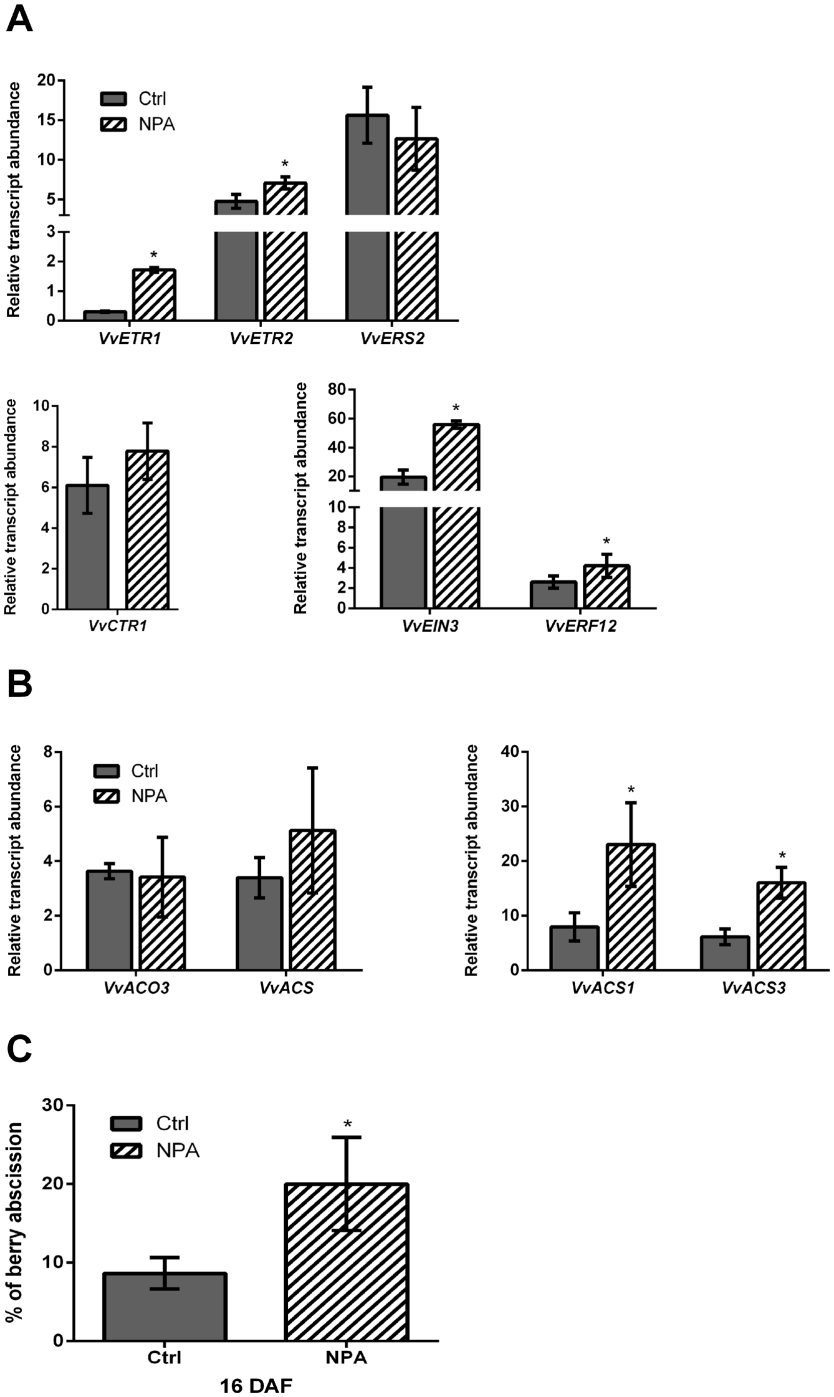
Transcript abundance of ethylene perception- and biosynthesis-related genes in “first berries” impaired in polar auxin transport. (A) Effect of blocking polar auxin transport at 14 DAF using 50 µM N-1-naphthylphthalamic acid (NPA) on transcript abundance of *VvETR1, VvETR2, VvERS2, VvCTR1, VvEIN3, VvERF12* genes assessed 2 days post treatment (DPT), at 16 DAF. (B) Effect of blocking polar auxin transport at 14 DAF using 50 µM NPA on transcript abundance of *VvACO3, VvACS, VvACS1* and *VvACS3* genes assessed 2 DPT, at 16 DAF. Transcript abundance is relative to the mean expression of the constitutive genes *VvUBI1* and *VvGPDH* (for more details, see Material and Methods section). (C) Effect of blocking polar auxin transport at 14 DAF on abscission using 50 µM NPA assessed 2 DPT, at 16 DAF. Treatment was done immediately after berry number per cluster was registered, and remaining berries were registered for percentage (%) of berry abscission estimation (for berry number see Table 2). Only “first berries” were analyzed. Asterisk indicates that differences are significant between NPA-treated and control berries. Significance was assessed using p value <0.05 (n = 3). Error bars are STERR±.

Low transcript abundance of genes involved in ethylene perception and biosynthesis was detected in “first berries” compared with “last berries” (Figure 3), correlated with low abscission rate and high percentage of polar auxin transport (Figure 2). Thus, we investigated whether auxin transport inhibition in “first berries” results in an increase in abscission, under the assumption that ethylene perception should be increased in response to impaired polar auxin transport. For this, first berries were treated with the auxin transport inhibitor, N-1-naphthylphthalamic acid (NPA), and changes in the transcript abundance of perception- and biosynthesis-related genes and in the abscission rate were assessed. It was found that relative transcript abundance of ethylene perception-related genes, *VvETR1*, *VvETR2*, *VvEIN3*, and *ERF12* was significantly increased in NPA-treated “first berries” compared to untreated “first berries”, with the transcript abundance of *VvCTR1* and *VvERS2* genes not affected by the treatment (Figure 5A). Auxin transport impairment also affected transcript relative levels of ethylene biosynthesis-related genes, with *VvACS1* and *VvACS3* induced by the NPA treatment (Figure 5B). As shown in Figure 5C, polar auxin transport impairment caused by NPA treatment, resulted in a slight but significant increase in abscission percentage, indicating that polar auxin transport across the berry is required to reduce abscission. Altogether these results suggest that the ability of “first berries” to persist in a cluster is based on the capacity to sustain polar transport of auxin, which is associated with changes in the expression of genes involved in ethylene biosynthesis and perception.

## Discussion

It is well known that a polar auxin transport dependent ethylene sensitization mechanism controls fruit abscission [1]. However, the molecular basis of this mechanism, regarding polar auxin transport and sensing, and induction of ethylene perception, is beginning to be unraveled. Recent work in tomato fruits has shed light into genes involved in the AZ formation [7]. It was shown that auxin depleted fruitlets were more prone to abscise due to a decrease in polar auxin transport capacity. This was related to an up-regulation of genes involved in ethylene biosynthesis and perception, as well as an alteration in the expression of auxin and ethylene-related transcription factors in auxin depleted fruitlets. In sum, auxin and ethylene were found to be important for abscission, controlling AZ formation. However, in this work the natural ability of some fruitlets to persist and the propensity of others to abscise were not assessed. Recently, comparisons at the molecular level between small abscising and big persisting apple fruitlets were performed [16]. In order to clearly differentiate these two types of fruits, some plants were treated to induce fruitlet abscission while others were manipulated to prevent abscission of big fruits. Differences in the expression of genes belonging to ethylene, abscisic acid and gibberellin pathways were found. However, it was not evaluated whether changes in polar auxin transport could explain the observed differences, despite it is well known that this is associated with fruit dominance [1].

In the present work, we compared abscising and persisting young grapevine berries, which were differentiated by the order they started their development, *i. e.* the day the flowers opened. To our knowledge, this approach has not been utilized previously for studying differences in abscission between fruits. It is important to mention that "first berries" and "late berries" were analyzed at the same developmental stage. It is also worth noticing that the comparisons between berries were done within the clusters and hence, all the categories that were analyzed received similar nutrient and hormone input from the plant and were under the same genetic background and environmental influence.

In this work it was found that flowers that opened the first day developed into bigger and more persistent berries than those derived from flowers that opened the following days (Figure 2A, Figure 2B). This suggests that “first berries” are “dominant” over “late berries”. It is possible to hypothesize that one of the features involved in this dominance status could be auxin transport. As shown in this work, polar auxin transport is important for preventing abscission, since its impairment in “first berries” causes berry drop and also changes in ethylene-related gene expression (Figure 5). The ability to persist of “first berries” during the abscission period was correlated with high polar auxin transport, while abscission in “late berries” was correlated with decreased auxin export (Figure 2C). So, it is possible that in “late berries” auxin retention within the fruit or the lack of a constant auxin flux through the pedicel could be triggering abscission. It is worth to mention that at 14 DAF polar auxin transport is lower than at 10 DAF and there are no significant differences between first and late formed berries regarding auxin export. The explanation for this could be that in cv Perlon the abscission process at this time is ending, as previously reported in cv Concord [20], and therefore polar auxin transport at 14 DAF could be less required than at 10 DAF.

High polar auxin transport was shown to be correlated with reduced transcript abundance of some ethylene biosynthesis- and perception-related genes in “first berries” compared to “last berries” (Figure 3). In the case of *VvCTR1* gene, its gene induction in “last berries” was not expected, since it is a putative negative regulator of the ethylene pathway, therefore increased transcript abundance should be associated with lower ethylene response. A possible explanation is that there is a feedback mechanism resulting in the induction of this gene in order to dampen the ethylene response. This makes sense, considering that abscission is a transient process and once it has occurred the ethylene response must be shut down. In grapevine berries treated with ethylene during ripening it is also observed an increase in *VvCTR1* transcripts [29], suggesting a role for this gene in a damping mechanism during this stage. We also observed an increase in the transcript abundance of two putative ethylene receptors, *VvETR1* and *VvERS2*, which are also putative negative regulators of the ethylene response. Although in theory, increased levels of receptor should result in a decrease in ethylene sensitivity, in all the ethylene responses observed only increases in receptor transcript abundance have been reported [30]. This can also be interpreted as a damping mechanism, as in the case of *VvCTR1*. Grapevine berries treated with ethylene at the onset of ripening also show an increase in the transcript abundance of these putative receptor genes [29]. In peach (*Prunus persica*) and apple (*Malus domestica*) fruits, there are differences in the response to 1-MCP, a strong antagonist of ethylene for receptor binding sites. While this compound delays ripening in apple, it has a limited effect on slowing ripening in peach. Interestingly, these differences were correlated with different expression patterns of *ETR1*, *ERS1* and *CTR1* genes, which were unaffected by 1-MCP treatment in peach, while they were down-regulated in apple [31], suggesting that the expression level of these genes might be connected with the degree of the ethylene response. In this regard, it is possible to hypothesize that 1-MCP is effective in delaying ripening in apple due to the inhibition of the ethylene response, and hence inactivation of damping mechanisms should occur as a consequence. This might explain the decrease in *ETR1*, *ERS1* and *CTR1* gene expression in apple, which are putative negative regulators. In contrast, the limited effect of 1-MCP on ripening in peach might explain why the expression of these genes does not change significantly. Nevertheless, more investigation is needed in order to unravel the connection between the expression of these genes and the sensitivity to ethylene, considering that there are several factors involved in its regulation. Finally, there was an increase in the transcript abundance of the putative transcription factors, *VvEIN3* and *VvERF12* in “last berries” compared to “first berries” (Figure 3A). In apple fruits, 1-MCP treatment results in down-regulation of several *EINs* and *ERFs* genes [32], suggesting a positive correlation between the expression of these genes and the ethylene response. It is worth to mention that also post-transcriptional regulation could have a role in the control of ethylene sensitivity by regulating the abundance of EIN3 protein. In this regard, it has been reported that ubiquitination of AtEIN3 mediated by Arabidopsis F-box proteins EBF1 and EBF2 represses ethylene response, resulting in growth promotion [33].

The transcript abundance of the putative ethylene biosynthetic genes, *VvACS1* and *VvACS3*, was higher in “last berries” compared to “first berries” (Figure 3B), supporting the idea of enhanced ethylene response in “last berries”. In apple, reduced expression of *ACS1*, *ACO1* and *ACO2* occurs when ripening is inhibited by 1-MCP treatment, suggesting that when the ethylene response is affected the expression of genes involved in the production of ethylene decreases [32]. In line with this idea, higher expression of putative ethylene biosynthetic genes might be a consequence of a more activated ethylene response in “last berries”. This is consistent with higher ethylene content measured in “last berries” compared with “first berries” (Figure 4). In summary, it is possible to suggest that if an increase in ethylene sensitivity in “last berries” is indeed occurring, then several ethylene responses might be activated, and one of these could be the biosynthesis of ethylene. Supporting this idea, an amplification mechanism has already been reported [26]–[28].

At this point it is worth to mention that “last berries” have higher area/volume ratio, so that differences in the transcript abundance could be due to skin-specific gene expression. In order to determine whether the analyzed genes are expressed preferentially in the skin or in the flesh, data of transcriptomic studies performed on grapevine berry tissues [34], [35] were analyzed. It was found that *EIN3* gene and several *ERF* genes were up-regulated in the skin [34], which suggests that higher levels of *VvEIN3* and *ERF12* transcripts found in “last berries” (Figure 3A) could be due, at least in part, to their enrichment in the skin. Conversely, an *ACS* coding transcript showed higher abundance in the flesh at pre-veraison [34]. If its expression only depends on the tissue type it would be expected lower levels of *VvACS* transcripts in “last berries”. However, *VvACS* did not change between “first berries” and “last berries”, and *VvACS1* and *VvACS3* transcript abundance was higher in “last berries” (Figure 3B), suggesting that flowering time could be a relevant factor influencing their expression. On the other hand, an *ETR2* gene and several *ACO* genes were up-regulated in the skin [34]. However, there were no differences in the accumulation of their transcripts between “first berries” and “last berries”, suggesting that flowering time could control the expression of these genes. In another transcriptomic study, an ethylene receptor gene was similarly expressed in skin and pulp [35]. Regarding the *CTR1* gene, its transcript abundance was higher in the skin [34] or similar in the skin and in the flesh [35], hence it is not clear whether skin-specific gene expression influences the transcript abundance of this gene. In summary, tissue-specific gene expression may account, at least in part, for differences in expression between “first berries” and “last berries”, but it does not exclude flowering time as an important factor underlying expression variation of some of the genes analyzed between berry categories.

Auxin transport inhibition in “first berries” resulted in increased transcript abundance of at least some ethylene biosynthesis- and perception-related genes (Figure 5), which was similar to that of “last berries” (Figure 3). This suggests that the transcript abundance of these genes is regulated by the auxin flux. Such is the case of *VvETR1*, *VvEIN3*, *VvERF12*, *VvACS1* and *VvACS3*, whose expression was high in “last berries” and also in NPA-treated “first berries”, where ethylene sensitivity is supposed to be increased. One noticeable exception was the gene coding for a putative receptor, *VvERS2*. This gene was induced in “last berries” compared to “first berries” (Figure 3A), but NPA treatment did not affect its transcript abundance (Figure 5A), suggesting that this gene is under the control of other mechanisms that are not sensitive to auxin flux sensing. The phytohorme gibberellin plays a central role during initial fruit development by triggering flower-to-fruit conversion and increasing fruit size [36]. In grapevine, it is well known the use of this hormone as a thinning compound when applied at bloom or post-bloom [37], therefore gibberellin could be underlying the differences in *VvERS2* expression between “first berries” and “last berries” at 12 DAF, considering that the highest levels of bioactive gibberellin have been reported around this time in grapevine berries [38].

**Figure 6:**
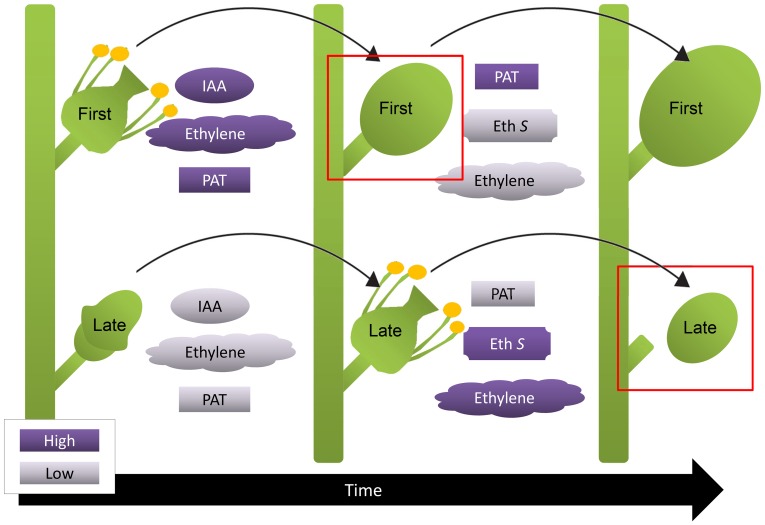
Proposed model for explaining differences in abscission between “first berries” and “late berries”. Flowering is associated with IAA and ethylene content increase, so flowers that open first may have higher content of these hormones than unopened flowers. IAA activates polar auxin transport (PAT), as shown in many plant species, so we propose that flowers that will originate “late berries” may have lower PAT than flowers that will originate “first berries”, and as a consequence “late berries” may have higher ethylene sensitivity (Eth S), which is associated with the activation of ethylene responses, such as the abscission zone formation. “First berries” should be unaffected by ethylene since they have high PAT and this might explain why abscission rate is lower in “first berries” than in “late berries”, when they are compared at the same developmental stage (red outlined boxes).

The present work demonstrates that “first berries” developed earlier and abscised less than “late berries”, and that these variations were related to auxin transport capacity. However, there were no significant differences between “late berries”. Hence, we hypothesized that berries originated from flowers that opened the first day exert a strong negative effect over the rest, while these less developed berries exert such a negative effect neither between them nor over “first berries”. This negative effect could be achieved by the release of negative signals by first developed flowers or berries. It is well known that after pollination there is an ethylene burst, as shown in other plant models [39], so we hypothesize that ethylene produced by flowers that open first is perceived by the flowers that are starting to open. Flowers also increase auxin levels in response to pollination, as it has been previously shown in tomato [40]. As opened flowers contain more auxin than still closed flowers and considering that auxin activates its own transport [41], [42], flowers that open first should quickly start to transport auxin toward the pedicels. Auxin export from the fruit has already been reported [9], [11]. Auxin transport activation in first flowers and berries could be associated with a decrease in ethylene sensitivity (Figure 6). On the other hand, ethylene represses polar auxin transport, as previously shown [43]–[46], thus ethylene might maintain polar auxin transport inactivated in “late berries”. Differences in auxin transport between fruits have already been reported in cherry (*Prunus avium*), which correlates with their abscission rate [9]. Interestingly, an amplification mechanism where ethylene activates its own biosynthesis has been described [47], [48]. Taking into account this positive feedback mechanism, it may be suggested that ethylene perceived by “late berries”, where ethylene perception and signaling elements are more abundant, would even more increase its own concentration compared to “first berries”. In summary, we propose that early in berry development, *i. e.* few days after pollination, “first berries” have high polar auxin transport and low ethylene sensitivity, while the rest of the flowers and berries have low polar auxin transport and hence are sensitive to ethylene produced by themselves and by “first berries” (Figure 6). Furthermore, ethylene might in turn inhibit polar auxin transport, as previously shown [43]–[46], explaining why polar transport of auxin is lower in “late berries” compared to “first berries” around 10 DAF, in concomitance with higher ethylene content (Figure 2C, Figure 4).

This work provides evidence showing the occurrence of variations in the ability to avoid early abscission in grapevine fruits, possibly through modulation of ethylene response by polar auxin transport. We analyzed whole berries since signals generated in them could be necessary for controlling molecular events occurring in the AZ. Differences between first developed berries and “last berries” regarding polar auxin transport, ethylene-related transcript abundance and ethylene content were observed and correlated to abscission variations between berries. Interestingly, when polar auxin transport was blocked in “first berries” they were more prone to abscise, indicating that auxin export is required for maintaining low abscission rate in these berries. To our knowledge, this is the first time that differences in abscission between first originated fruits and late fruits are investigated in terms of polar auxin transport and ethylene-related gene expression.

## Materials and Methods

### Experimental design, berry tagging and sampling

Grapevine plants (*Vitis vinifera* L. cv Perlon) were selected from an experimental field located in the Curacaví Valley, Chile (GPS coordinates 33° 24′02.48″S, 71° 03′18.40″W), during the 2012/2013 growing season. The field trial did not require special permission and field studies did not involve endangered or protected species.

Grapevine clusters bearing approximately 500 flowers were used. Nine clusters were randomly selected from three plants in order to study naturally occurring variations between berries. Additionally, twelve clusters were randomly selected from three plants for assessing the effect of blocking polar auxin transport, with six clusters treated with 50 µM N-1-naphthylphthalamic acid (NPA) in a lanoline/vaseline (1∶3) mix and six clusters treated with control solution (lanoline/vaseline (1∶3) mix). Treatments with NPA were performed at 14 days after flowering (DAF) and berries were collected 2 days post treatment (DPT), at 16 DAF. In all cases the entire berry, including its pedicel, was covered with a thin layer of lanoline/vaseline (1∶3) mix.

In order to differentiate first and late formed berries according to the flowering order, a flower tagging system was adapted from [49]. In brief, opened flowers in a cluster were tagged daily with different colored threads that were knotted around the pedicel, using one color per day. This allowed to distinguish four categories of berries per cluster. Berries derived from flowers that opened the day that flowering started, *i. e.* day 1, correspond to “berry category one (1)” named as “first berries”; berries derived from flowers that opened the second, third and fourth day of flowering, correspond to berry categories two (2), three (3) and four (4) respectively, named as “late berries”. Berries belonging to category 4 are also named as “last berries”, since a negligible number of flowers opened the days that followed day 4.

For berry sampling three biological replicates were used, where a biological replicate is composed of a pool of berries. For polar auxin transport and berry volume estimation, 6 berries per cluster were selected. For RNA extraction and ethylene measurements 0.5 g of berries per cluster were used. For abscission rate estimation, berries tagged with same color within a cluster were registered. Sampling was performed at the same hour of the day (between 10 am and 2 pm).

### Volume and abscission estimation

Berry volume was assessed at 10 and 14 DAF for each of the four berry categories. For berry volume estimation, the transversal and longitudinal diameters (TD and LD, respectively) were measured using a caliper. Next, average diameters were used for berry volume estimation according to the equation 1:
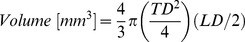
(1)


For berry abscission estimation at 12 DAF, berry number per category within each cluster was registered by counting the same color threads that were tied to the pedicels. Berry number register was performed at 8 and 12 DAF, with berry number at 8 DAF set as 100%. Then, relative abscission percentage (%) was estimated according to equation 2: 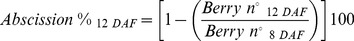
(2)


Where *Berry n°* corresponds to the same color tagged berries registered within a cluster. Two technical replicates and three biological replicates were used. To estimate berry abscission % in NPA experiment, berry number within each cluster was registered at 14 and 16 DAF in NPA-treated and control berries. Equation (2) was used, with berry number at 16 DAF set as 100%. The values of all replicates are shown in Tables 1 and 2.

**Table 1:** Berry number per cluster at 8 and 12 DAF in categories 1, 2, 3 and 4.

			8 DAF						12 DAF			
	R1,T1	R1,T2	R2,T1	R2,T2	R3,T1	R3,T2	R1,T1	R1,T2	R2,T1	R2,T2	R3,T1	R3,T2
**1**	52	50	149	143	137	143	49	48	133	139	138	124
**2**	83	93	92	98	118	116	70	66	78	82	101	102
**3**	95	103	113	119	184	178	74	72	89	87	158	164
**4**	66	64	95	106	143	137	35	36	62	64	99	92

R1–R3, biological replicates. T1–T2, technical repetitions.

**Table 2:** Berry number per cluster at 14 and 16 DAF in NPA (N) and control (C) treatments performed at 14 DAF.

	14 DAF						16 DAF					
	R1,T1	R1,T2	R2,T1	R2,T2	R3,T1	R3,T2	R1,T1	R1,T2	R2,T1	R2,T2	R3,T1	R3,T2
**N**	47	45	85	86	56	57	41	38	75	72	39	38
**C**	53	51	133	136	126	128	51	48	118	120	112	110

R1–R3, biological replicates. T1–T2, technical repetitions.

### Polar auxin transport measurements

Tritium-labeled Indole-3-acetic acid ([5-^3^H]IAA, specific activity 50.55 TBq mmol^−1^, concentration 1 mCi mL^−1^, American Radiolabeled Chemicals Inc.) was diluted 1∶10 times in pure ethanol, to a final [5-^3^H]IAA concentration of 4 µM. Polar auxin transport experiment was adapted from [9], to measure auxin transport across the berries instead of the pedicels. In brief, berries were excised at the distal end of the pedicel for sampling. Next, the pedicels were removed from the berries under deionized water, using a sharp razor blade, and a 0.5 mm diameter hole was made in the apical surface of the berries, for the [5-^3^H]IAA drop. Then, each berry was put with its basal side in contact with a receiver agar disc (1.5% (w/v) agar-agar (Merk), 0.2% (w/v) MES (Sigma-Aldrich), in a final volume of 300 µL at pH 5.5) placed on a 24 well tissue culture plate (Sigma Aldrich). After adding a 0.2 µL drop of [5-^3^H]IAA into the hole at the apical surface of each berry, the plate was covered and berries were incubated for 8 hours at 22°C. A humid paper towel was fixed to the cover, in order to avoid dehydration. After incubation, agar discs and berries were homogenized in 2 mL of 80% methanol and kept at 4°C with overnight agitation. The accumulated radioactivity in the receiver agar discs was determined by radioactive scintillation counting of disintegrations per minute (DPM) in a liquid scintillation analyzer (Beckman Ls6500), using a vial containing 3 mL of OptiPhase HiSafe 3 (Perkin-Elmer), as liquid scintillation cocktail. Results were expressed as percentage (%) of polar auxin transport and were corrected by volume and contact surface in order to cancel size differences, according to the equation 3, adapted from [50]:
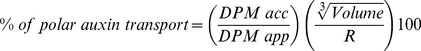
(3)


Where *DPM acc* is the accumulated radioactivity in the receiver agar discs after 8 hours of incubation and *DPM app* is the total applied radioactivity, and equals the radioactivity in the receiver agar discs plus the radioactivity in the berries after 8 hours of incubation. *Volume* is obtained using berry volume equation (1), and *R* is the radius of the contact surface in a transversal cut. For acropetal transport assessment, the radioactive drop was added to the berry-pedicel junction and the berry was placed with its apical surface in contact with the agar disc. In order to determine whether auxin transport is polar, 50 µM NPA in a lanoline:vaseline (1∶3) mix, was added *in planta* 24 hours prior to the auxin transport experiment. Acropetal and NPA controls were around 30% and 50% of basipetal transport, respectively (unpublished observations), similar to previously reported in Arabidopsis seedlings [50]. Basipetal transport quantifications were not corrected by these values.

### Ethylene content measurements

Immediately after sampling, berries were enclosed in 2 mL tubes with screw lids, one tube per replicate. Tubes were maintained at 22°C for three hours and then heated at 100°C for 90 min in order to release remaining ethylene out of the berries [4]. After cooling, 1 mL of air sample was withdrawn from the headspace using a syringe, and ethylene content was quantified using a Photovac 10s Plus gas chromatograph (Photovac, Markham), equipped with a photoionization detector. Ethylene content in a tube with no vegetal material was measured and set as zero for normalization.

### RNA extraction and cDNA synthesis

Total RNA was extracted from 0.5 g of a pool of seven to ten berries selected from a bunch. Extraction was performed using a modified CTAB-Spermidine extraction method, as previously described [51]. RNA was treated with Ambion TURBO DNA-free DNase, according to the manufacturer's instructions. RNA concentration and purity was assessed using a Thermo Scientific NanoDrop ND-1000 spectrophotometer, where A260/A280 ratio values between 1.8 and 2.0 and A260/A230 ratio values >2.0 were considered acceptable. RNA integrity was evaluated by performing routine electrophoresis in a denaturing MOPS buffer/formaldehyde gel. For all samples two sharp bands (28S rRNA and 18S rRNA) were observed. For cDNA synthesis, total RNA (1.5 µg) was reverse transcribed with random hexamer primers using SuperScript II reverse transcriptase (Invitrogen), according to the manufacturer's instructions.

### Quantitative analysis of transcript abundance

Real time RT-qPCR was performed using a MX3000P detection system (Stratagene), as previously described [52], and the SensiMix Plus SYBR commercial kit (Quantace) was utilized, according to the manufacturer's instructions. A fragment of the grapevine *VvGPDH* gene (*VvGLYCERALDEHYDE-3-PHOSPHATE DEHYDROGENASE*, GI: 359491598) [53] and a fragment of the grapevine *VvUBI1* gene (*VvUbiquitin1*, TC53702, TIGR database) [54] were utilized as housekeepings genes. In order to estimate relative transcript abundance values, a ratio between the expression of the gene of interest (*GOI*) and the geometric mean of the expression of the housekeeping genes, *VvGPDH* and *VvUBI1*, was generated according to the equation 4: 
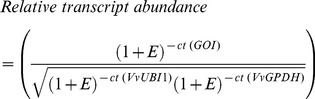
(4)


Where E corresponds to each primer amplification efficiency value. Ct values, relative quantities and normalized relative quantities for *VvGPDH* and *VvUBI1* in categories 1 and 4 and in NPA and control treatments are given in Table S1. qPCR conditions, standard quantification curves for each gene and E values were conducted according to [51]. All experiments were performed using three biological replicates (three bunches coming from three different plants) and three technical replicates.

RT-qPCR primer sequences for *VvETR1* (*Vitis vinifera ethylene receptor 1-like*, GenBank accession: AAF63755.1; V1 annotation: VIT_19s0093g00580), *VvETR2* (*Vitis vinifera ethylene receptor 2-like*, GenBank accession: CAN84042.1; V1 annotation: VIT_05s0049g00090), *VvCTR1* (*Vitis vinifera serine/threonine-protein kinase CTR1-like*, GenBank accession: CAO15968.1), *VvERS2* (*Vitis vinifera ethylene receptor 2*, GenBank accession: XP_002272649.1; V1 annotation: VIT_07s0005g00850) and *VvEIN3* (*Vitis vinifera protein ETHYLENE INSENSITIVE 3-like*, GenBank accession: XM_002276380; V1 annotation: VIT_06s0009g01380) were obtained from [29]. Primers for *VvERF12* (unnamed protein product; GenBank accession: CBI35177.3; V1 annotation: VIT_14s0081g00520) were designed using Primer3plus [55]. Primers for *VvACO3* (*Vitis vinifera 1-aminocyclopropane-1-carboxylate oxidase 3-like*, GI: 359485521), *VvACS* (*Vitis vinifera 1-aminocyclopropane-1-carboxylate synthase-like*, GI: 359474170), *VvACS1* (*Vitis vinifera 1-aminocyclopropane-1-carboxylate synthase 1-like*, GI: 359489806), *VvACS3* (*Vitis vinifera 1-aminocyclopropane-1-carboxylate synthase 3-like*, GI: 359497339) were designed using Primer-BLAST tool available on NCBI webpage. Amplicon size was <200 pb. PCR products were excised from an agarose gel, purified using Qiaex II (Qiagen) and sequenced. Primers sequences are listed in Table 3. Arithmetic mean of *VvUBI1* and *VvGPDH* relative transcript abundance was 10^3^ times the relative transcript abundance of *VvETR1*, *VvETR2*, *VvERS1*, *VvEIN3* and *VvERF12*; 10^6^ times that of *VvCTR1*, *VvACS1* and *VvACS3*, and 10^2^ times that of *VvACO3* and *VvACS*.

**Table 3:** RT-qPCR primers.

Name	Primer sequences (5′ – 3′)	References
*VvUBI1*	F:TCTGAGGCTTCGTGGTGGTA	[48]
	R:AGGCGTGCATAACATTTGCG	
*VvGPDH*	F:TTGGCATTGTGGAGGGTCTT	[47]
	R:TTCCACCTCTCCAGTCCTTCA	
*VvETR1*	F:AGAACACCTATGCATGCCATCA	[29]
	R:CTGCTCTTTAGGATGGCTTCAAC	
*VvETR2*	F:CCAAAAGCATGGCTCTCGTT	[29]
	R:TGGTTCAGAAATGTTGATTCCAA	
*VvERS2* *	F:GCCCCTCACTTTCAATCCAA	[29]
	R:TGGACTCGCCATTTGTAAACG	
*VvCTR1*	F:GCACAAACCTGGTGCAAGAGA	[29]
	R:TCATGCCCTTGGCCACAT	
*VvEIN3*	F:CTGGTGGGAGTGGATCTTTTG	[29]
	R:CCTATCTCTGGCTCCTACGCC	
*VvERF12*	F:TTGCAGCGGAGATTAGAGATCC	This study
	R:AAATTCGTCTTGGCCTTGGG	
*VvACO3*	F:CCGAGCCCACACTGATGCCG	This study
	R:TGGAGTGGCGCATTGGAGGA	
*VvACS*	F:ACCCGTTGGGGACAGTTCTGG	This study
	R:GCCGGGTGAGCTGAAGACGG	
*VvACS1*	F:GCCAGGAGGAGGCAGAGGCA	This study
	R:GCGCGGACGAGTGGGGAATG	
*VvACS3*	F:TCCTGGGATGGGAGGAATACGAGAA	This study
	R:TGGGTTTTGTGCGAGCCAGGA	

*ERS1 in the original paper [29].

### Statistical analysis

For berry volume, abscission percentage, polar auxin transport, ethylene content and transcript abundance measurements, three biological replicates were used. Tukey's media comparison analyses were performed in order to compare different conditions. For all the analyses, statistical significance was assessed using p value <0.05.

## Supporting Information

Table S1 RT-qPCR Ct values for *VvUBI1* and *VvGPDH* genes.
(PDF)

## References

[1] Bangerth F (2000) Abscission and thinning of young fruit and their regulation by plant hormones and bioregulators. Plant Growth Reg 31: 43–59.

[2] Bennett M, Bellini C, Van Der Straeten D (2005) Integrative biology: Dissecting cross-talk between plant signaling pathways. Physiol Plant 123: 109.

[3] Brown KM (1997) Ethylene and abscission. Physiol Plant 100: 567–76.

[4] Hilt C, Bessis R (2003) Abscission of grapevine fruitlets in relation to ethylene biosynthesis. Vitis 42: 1–3.

[5] Iannetta PPM, Wyman M, Neelam A, Jones C, Taylor MA, et al (2000) A causal role for ethylene and endo-β-1,4-glucanase in the abscission of red-raspberry (*Rubus idaeus*) drupelets. Physiol Plant 110: 535–543.

[6] Ruperti B, Bonghi C, Tonutti P, Ramina A (1998) Ethylene biosynthesis in peach fruitlet abscission. Plant Cell Environ 21: 731–737.

[7] Meir S, Philosoph-Hadas S, Sundaresan S, Selvaraj KSV, Burd S, et al (2010) Microarray analysis of the abscission-related transcriptome in the tomato flower abscission zone in response to auxin depletion. Plant Physiol 154: 1929–1956. 20947671 10.1104/pp.110.160697 PMC2996037

[8] Taylor JE, Whitelaw CA (2001) Signals in abscission. New Phytol 151: 323–39.

[9] Else M, Stankiewicz-Davies A, Crisp C, Atkinson C (2004) The role of polar auxin transport through pedicels of *Prunus avium* L. in relation to fruit development and retention. J Exp Bot 55: 2099–109. 15310825 10.1093/jxb/erh208

[10] Blanusa T, Else MA, Atkinson CJ, Davies W (2005) The regulation of sweet cherry fruit abscission by polar auxin transport. Plant Growth Reg 45: 189–98.

[11] Serrani J, Carrera E, Ruiz-Rivero O, Gallego-Giraldo L, Pereira-Peres L, et al (2010) Inhibition of auxin transport from the ovary or from the apical shoot induces parthenocarpic fruit-set in tomato mediated by gibberellins. Plant Physiol 153: 851–62. 20388661 10.1104/pp.110.155424 PMC2879769

[12] Sexton R, Roberts JA (1982) Cell biology of abscission. Annu Rev Plant Physiol 33: 133–62.

[13] Meir S, Hunter DA, Chen JC, Halaly V, Reid MS (2006) Molecular changes occurring during acquisition of abscission competence following auxin depletion in *Mirabilis jalapa* . Plant Physiol 141: 1604–16. 16778017 10.1104/pp.106.079277 PMC1533941

[14] Dal Cin V, Danesin M, Boschetti A, Dorigoni A, Ramina A (2005) Ethylene biosynthesis and perception in apple fruitlet abscission (*Malus domestica* L. Borkh). J Exp Bot 56: 2995–3005. 16203755 10.1093/jxb/eri296

[15] Gruber JD, Bangerth F (1990) Diffusible IAA and dominance phenomena in fruits of apple and tomato. Physiol Plant 79: 354–358.

[16] Botton A, Eccher G, Forcato C, Ferrarini A, Begheldo M, et al (2011) Signaling pathways mediating the induction of apple fruitlet abscission. Plant Physiol 155: 185–208. 21037112 10.1104/pp.110.165779 PMC3075760

[17] Pratt C (1971) Reproductive anatomy in cultivated grapes: A review. Am J Enol Vitic 22: 92–109.

[18] Coombe B (1960) Relationship of growth and development to changes in sugars, auxins and gibberellins in fruit of seeded and seedless varieties of *Vitis vinifera* L. Plant Physiol 35: 241–50. 16655336 10.1104/pp.35.2.241 PMC405950

[19] Ojeda H, Deloire A, Carbonneau A, Ageorges A, Romieu C (1999) Berry development of grapevines: relations between the growth of berries and their DNA content indicate cell multiplication and enlargement. Vitis 38: 145–50.

[20] Intrigliolo DS, Lakso AN (2009) Berry abscission is related to berry growth in “*Vitis labruscana*” Concord and “*Vitis vinifera*” Riesling. Vitis 48: 53–54.

[21] Pratt C (1973) Reproductive systems of Concord and two sports (*Vitis labruscana* Bailey). J Am Soc Hortic Sci 98: 489–496.

[22] Bessis R, Fournioux JC (1992) Zone d'abscission et coulure dela vigne. Vitis 31: 9–21.

[23] Bessis R, Charpentier N, Hilt C, Fournioux JC (2000) Grapevine fruit set: Physiology of the abscission zone. Aust J Grape Wine Res 6: 125–30.

[24] Böttcher C, Keyzers R, Boss P, Davies C (2010) Sequestration of auxin by the indole-3-acetic acid-amido synthetase GH3-1 in grape berry (*Vitis vinifera* L.) and the proposed role of auxin conjugation during ripening. J Exp Bot 61: 3615–25. 20581124 10.1093/jxb/erq174

[25] Constantini E, Landi L, Silvestroni O, Pandolfini T, Spena A, et al (2007) Auxin synthesis-encoding transgene enhances grape fecundity. Plant Physiol 143: 1689–94. 17337528 10.1104/pp.106.095232 PMC1851826

[26] Barry CS, Llop-Tous MI, Grierson D (2000) The regulation of 1-aminocyclopropane-1-carboxylic acid synthase gene expression during the transition from system-1 to system-2 ethylene synthesis in tomato. Plant Physiol 123: 979–86. 10889246 10.1104/pp.123.3.979 PMC59060

[27] Llop-Tous I, Barry CS, Grierson D (2000) Regulation of ethylene biosynthesis in response to pollination in tomato flowers. Plant Physiol 123: 971–8. 10889245 10.1104/pp.123.3.971 PMC59059

[28] Petruzzelli L, Coraggio I, Leubner-Metzger G (2000) Ethylene promotes ethylene biosynthesis during pea seed germination by positive feedback regulation of 1-aminocyclopropane-1-carboxylic acid oxidase. Planta 211: 144–9. 10923715 10.1007/s004250000274

[29] Chervin C, Deluc L (2010) Ethylene signaling and transcription factors over the grape berry development: gene expression profiling. Vitis 49: 129–36.

[30] Ciardi J, Klee H (2001) Regulation of ethylene-mediated responses at the level of the receptor. Ann Bot 88: 813–822.

[31] Dal Cin V, Rizzini FM, Botton A, Tonutti P (2006) The ethylene biosynthetic and signal transduction pathways are differently affected by 1-MCP in apple and peach fruit. Postharvest Biol Tech 42: 125–133.

[32] Yang X, Song J, Campbell-Palmer L, Fillmore S, Zhang Z (2013) Effect of ethylene and 1-MCP on expression of genes involved in ethylene biosynthesis and perception during ripening of apple fruit. Postharvest Biol Tech 78: 55–66.

[33] Gagne JM, Smalle J, Gingerich DJ, Walker JM, Yoo SD, et al (2004) Arabidopsis EIN3-binding F-box 1 and 2 form ubiquitin-protein ligases that repress ethylene action and promote growth by directing EIN3 degradation. Proc Natl Acad Sci USA 101: 6803–6808. 15090654 10.1073/pnas.0401698101 PMC404126

[34] Lijavetzky D, Carbonell-Bejerano P, Grimplet J, Bravo G, Flores P, et al (2012) Berry flesh and skin ripening features in *Vitis vinifera* as assessed by transcriptional profiling. PLoS One 7: e39547. 22768087 10.1371/journal.pone.0039547 PMC3386993

[35] Grimplet J, Deluc LG, Tillett RL, Wheatley MD, Schlauch KA, et al (2007) Tissue-specific mRNA expression profiling in grape berry tissues. BMC Genomics 8: 187. 17584945 10.1186/1471-2164-8-187 PMC1925093

[36] De Jong M, Mariani C, Vriezen WH (2009) The role of auxin and gibberellin in tomato fruit set. J Exp Bot 60: 1523–32. 19321650 10.1093/jxb/erp094

[37] Dokoozlian NK, Peacock WL (2001) Gibberellic acid applied at bloom reduces fruit set and improves size of ‘Crimson Seedless’ table grapes. HortScience 36: 706–709.

[38] Pérez FJ, Viani C, Retamales J (2000) Bioactive gibberellins in seeded and seedless grapes: identification and changes in content during berry development. Am J Enol Vitic 51: 315–318.

[39] O'Neill SD (1997) Pollination regulation of flower development. Annu Rev Plant Physiol Plant Mol Biol 48: 547–74. 15012274 10.1146/annurev.arplant.48.1.547

[40] Mariotti L, Picciarelli P, Lombardi L, Ceccarelli N (2011) Fruit-set and early fruit growth in tomato are associated with increases in indoleacetic acid, cytokinin, and bioactive gibberellin contents. J Plant Growth Regul 30: 405–415.

[41] Nick P, Han MJ, An G (2009) Auxin stimulates its own transport by shaping actin filaments. Plant physiol 151: 155–167. 19633235 10.1104/pp.109.140111 PMC2736007

[42] Vieten A, Vanneste S, Wisniewska J, Benková E, Benjamins R, et al (2005) Functional redundancy of PIN proteins is accompanied by auxin-dependent cross-regulation of *PIN* expression. Development 132: 4521–4531. 16192309 10.1242/dev.02027

[43] Morgan P, Gausman H (1966) Effects of ethylene on auxin transport. Plant Physiol 41: 45–52. 16656230 10.1104/pp.41.1.45 PMC1086294

[44] Suttle JC (1988) Effect of ethylene treatment on polar IAA transport, net IAA uptake and specific binding of N-1-naphthylphthalamic acid in tissues and microsomes isolated from etiolated pea epicotyls. Plant Physiol 88: 795–9. 16666386 10.1104/pp.88.3.795 PMC1055663

[45] Lee JS, Chang WK, Evans ML (1990) Effects of ethylene on the kinetics of curvature and auxin redistribution in gravistimulated roots of *Zea mays* . Plant Physiol 94: 1770–5. 11537475 10.1104/pp.94.4.1770 PMC1077451

[46] Buer CS, Sukumar P, Muday GK (2006) Ethylene modulates flavonoid accumulation and gravitropic responses in roots of *Arabidopsis thaliana* . Plant Physiol 140: 1384–96. 16489132 10.1104/pp.105.075671 PMC1435817

[47] Arteca JM, Arteca RN (1999) A multi-responsive gene encoding 1-aminocyclopropane-1-carboxylate synthase (*ACS6*) in mature *Arabidopsis* leaves. Plant Mol Biol 39: 209–19. 10080689 10.1023/a:1006177902093

[48] Wang NN, Shih M-C, Li N (2005) The GUS reporter-aided analysis of the promoter activities of *Arabidopsis* ACC synthase genes *AtACS4*, *AtACS5*, and *AtACS7* induced by hormones and stresses. J Exp Bot 56: 909–20. 15699063 10.1093/jxb/eri083

[49] Friend A, Creasy GL, Trought M, Lang A (2003) Use of tagging to trace capfall and development of individual *Vitis Vinifera* L. cv. Pinot noir flowers. Am J Enol Vitic 54: 313–7.

[50] Liu X, Cohen JD, Gardner G (2011) Low-fluence red light increases the transport and biosynthesis of auxin. Plant Physiol 157: 891–904. 21807888 10.1104/pp.111.181388 PMC3192557

[51] Poupin MJ, Federici F, Medina C, Matus JT, Timmermann T, et al (2007) Isolation of the three grape sublineages of B-class MADS-box *TM6*, *PISTILLATA* and *APETALA3* genes which are differentially expressed during flower and fruit development. Gene 404: 10–24. 17920788 10.1016/j.gene.2007.08.005

[52] Dauelsberg P, Matus JT, Poupin MJ, Leiva-Ampuero A, Godoy F, et al (2011) Effect of pollination and fertilization on the expression of genes related to floral transition, hormone synthesis and berry development in grapevine. J Plant Physiol 168: 1667–74. 21497942 10.1016/j.jplph.2011.03.006

[53] Vega A, Gutiérrez RA, Peña-Neira A, Cramer GR, Arce-Johnson P (2011) Compatible GLRaV-3 viral infections affect berry ripening decreasing sugar accumulation and anthocyanin biosynthesis in *Vitis vinifera* . Plant Mol Biol 77: 261–274. 21786204 10.1007/s11103-011-9807-8

[54] Downey MO, Harvey JS, Robinson SP (2003) Synthesis of flavonols and expression of flavonols synthase genes in the developing grape berries of Shiraz and Chardonnay (*Vitis vinifera* L.). Aust J Grape Wine Res 9: 110–21.

[55] Untergasser A, Cutcutache I, Koressaar T, Ye J, Faircloth BC, et al (2012) Primer3–new capabilities and interfaces. Nucleic Acids Res 40: e115. 22730293 10.1093/nar/gks596 PMC3424584

