# Supplementary material for: Differential Behavior within a Grapevine Cluster: Decreased Ethylene-Related Gene Expression Dependent on Auxin Transport Is Correlated with Low Abscission of First Developed Berries
Source: PLoS One. 2014 Nov 3;9(11):e111258. doi: 10.1371/journal.pone.0111258 (PMC4218718; doi:10.1371/journal.pone.0111258)
Supplement: Table S1 — RT-qPCR Ct values for VvUBI1 and VvGPDH genes. (PDF) [file pone.0111258.s001.pdf]

Supplementary Table S1. RT-qPCR Ct values for *VvUBI1* and *VvGPDH* genes in berry category 1 and 4 at 12 DAF, and in NPA (N) and control (C) berries at 16 DAF. Three biological and three technical replicates were performed.

VvUBI1				*VvGPDH*			
**Replicates**	**Ct (dRn)**	**Replicates**	**Ct (dRn)**	**Replicates**	**Ct (dRn)**	**Replicates**	**Ct (dRn)**
1.1	19.67	N.1	19.7	1.1	19.98	N.1	19.56
1.1	19.69	N.1	19.84	1.1	19.84	N.1	19.61
1.1	19.68	N.1	19.66	1.1	19.8	N.1	19.47
1.2	21.86	N.3	19.33	1.2	20.82	N.2	19.53
1.2	21.04	N.3	19.48	1.2	20.96	N.2	19.4
1.2	21.21	N.3	19.61	1.2	21	N.2	19.43
1.3	20.46	N.2	19.42	1.3	19.6	N.3	19.18
1.3	20.65	N.2	19.48	1.3	19.92	N.3	19.19
1.3	20.74	N.2	19.26	1.3	19.87	N.3	19.39
4.1	20.37	C.1	21.12	4.1	20.1	C.1	20.37
4.1	20.16	C.1	21.11	4.1	20.1	C.1	19.51
4.1	20.49	C.1	21.12	4.1	20.1	C.1	19.98
4.2	19.55	C.2	21.14	4.2	19.46	C.2	20.52
4.2	19.65	C.2	21.25	4.2	19.33	C.2	21.24
4.2	19.61	C.2	21.17	4.2	19.29	C.2	21.28
4.3	19.5	C.3	21.08	4.3	19.49	C.3	19.83
4.3	19.35	C.3	21.14	4.3	19.61	C.3	20.04
4.3	20.11	C.3	21.22	4.3	19.64	C.3	19.29
